# 5-Aminoisoquinolinone, a PARP-1 Inhibitor, Ameliorates Immune Abnormalities through Upregulation of Anti-Inflammatory and Downregulation of Inflammatory Parameters in T Cells of BTBR Mouse Model of Autism

**DOI:** 10.3390/brainsci11020249

**Published:** 2021-02-17

**Authors:** Khaled Alhosaini, Mushtaq A. Ansari, Ahmed Nadeem, Saleh A. Bakheet, Sabry M. Attia, Khalid Alhazzani, Thamer H. Albekairi, Haneen A. Al-Mazroua, Hafiz M. Mahmood, Sheikh F. Ahmad

**Affiliations:** Department of Pharmacology and Toxicology, College of Pharmacy, King Saud University, Riyadh 11451, Saudi Arabia; kalhosaini@ksu.edu.sa (K.A.); muansari@ksu.edu.sa (M.A.A.); anadeem@ksu.edu.sa (A.N.); sbakheet@ksu.edu.sa (S.A.B.); attiasm@ksu.edu.sa (S.M.A.); Kalhazzani@ksu.edu.sa (K.A.); thalbekairi@ksu.edu.sa (T.H.A.); halmazroua@ksu.edu.sa (H.A.A.-M.); harshad@ksu.edu.sa (H.M.M.)

**Keywords:** autism, BTBR mice, 5-AIQ, cytokines, transcription factors, spleen and brain tissue

## Abstract

Autism spectrum disorder (ASD) covers a range of neurodevelopmental disorders involving impairments in communication and repetitive and stereotyped patterns of behavior and reciprocal social interaction. 5-Aminoisoquinolinone (5-AIQ), a PARP-1 inhibitor, has neuroprotective and anti-inflammatory effects. We investigated the influence of 5-AIQ-treatment in BTBR T+ Itpr3tf/J (BTBR) mice as an autism model and used flow cytometry to assess the effect of 5-AIQ on FOXP3, Helios, GATA3, IL-9, IL-10 and IL-17A production by CXCR6+ and CD4+ T cells in the spleen. We also confirmed the effect of 5-AIQ treatment on expression of FOXP3, Helios, GATA3, IL-17A, IL-10, and IL-9 mRNA and protein expression levels in the brain tissue by quantitative PCR and western blotting. Our results demonstrated that 5-AIQ-treated BTBR mice had significantly increased numbers of CXCR6+FOXP3+, CXCR6+IL-10+, and CXCR6+Helios+ cells and decreased numbers of CD4+GATA3+, CD4+IL-9+, and CD4+IL-17A+ cells as compared with those in untreated BTBR mice. Our results further demonstrated that treatment with 5-AIQ in BTBR mice increased expression for FOXP3, IL-10, and Helios, and decreased expression for GATA3, IL-17A, and IL-9 mRNA. Our findings support the hypotheses that 5-AIQ has promising novel therapeutic effects on neuroimmune dysfunction in autism and is associated with modulation of Treg and Th17 cells.

## 1. Introduction

Autism spectrum disorders (ASD) cover a range of common neurobehavioral disorders characterized by impairments in social interaction and verbal and nonverbal communication, and stereotyped patterns of behaviors and reciprocal social interaction [1]. Presently, the pathogenesis of ASD remains unclear, but immune dysfunction has been suggested as a major etiological component associated with ASD pathophysiology [2]. Furthermore, several genetic studies linked ASD with genes that are involved with various immune functions [3,4]. In addition, immunological abnormalities have been reported in children with ASD, specifically in the levels of inflammatory mediators and autoimmune responses [5,6]. There is strong evidence that disruption of cytokine levels has a significant role as a risk factor for several neurodevelopmental defects, including autism [7,8]. Previous studies showed that increased chemokine and cytokine levels were associated with increased aberrant behavior and impaired development [9,10]. Recently, we reported that dysregulation of inflammatory mediators and transcription factors’ expression are associated with the severity of ASD development [11,12], although the underlying mechanisms of this action remain elusive.

A range of immunomodulatory proteins may be involved in ASD. Regulatory T (Treg) cell differentiation and function are driven by the FOXP3 transcription factor, and the expression of FOXP3 has been shown to be decreased in individuals who develop neurologic diseases; for example, a decreased frequency of Treg cells has been found in children with ASD [13,14]. IL-10 is an anti-inflammatory cytokine produced by CD4+ Treg cells and can suppress inflammatory responses [15,16]. Helios is a transcription factor which binds to the FOXP3 promoter and triggers FOXP3 synthesis [17]. GATA3 is a transcription factor that is associated with inflammation and cytokine production [18,19] and is also involved in sympathetic neuron development [20]; upregulation of GATA3 expression is involved in the development of serotonergic neurons [21].

IL-9 is involved in the development of autoimmune and neuroinflammatory disorders [22,23], has an important immunoregulatory role in the progression of neurodevelopment [24], and is highly expressed in the central nervous system (CNS) [25]. IL-17A promotes sociability in the mouse model of neurodevelopmental disorder [26], and increased production of IL-17A in pregnant mothers can promote autism-like phenotypes in offspring [27,28]. Increased IL-17A signaling correlates with immune aberration in ASD [29]. Blocking of IL-17A expression was also shown to ameliorate ASD-like behaviors [26].

Poly(ADP-ribose) polymerase-1 (PARP)-1 can promote tumor growth and progression through DNA repair activity [30]. The therapeutic effect of a PARP-1 inhibitor on experimental animals has been shown to downregulate the inflammatory response [31]. A PARP-1 inhibitor was shown to significantly ameliorate lipopolysaccharide-induced neurobehavioral and neurochemical abnormality in mice [32]. In our previous study, we highlighted the beneficial effects of 5-aminoisoquinolinone (5-AIQ) on self-grooming, marble burying, and enhanced social interactions in BTBR T+ Itpr3tf/J (BTBR) mice in a mouse autism model, and also highlighted the contribution of Th1/Th22 cells [33].

The BTBR mouse autism model has become a standard method for the assessment of the efficacy of potential drugs that would target the autism disorder in clinical studies. BTBR mice demonstrate several behavioral characteristics including repetitive self-grooming, social deficits and impaired communication that may be relevant to autism. These factors are all relevant to the core symptoms of ASD [34]. In addition, aberrant immune responses have been observed in BTBR mice [35,36]. BTBR mice were characterized by lower levels of Foxp3^+^ and higher levels of RORγt^+^, T-bet^+^, and GATA-3^+^ production in CD4^+^ T cells [12]. C57BL/6 (C57) mice displayed higher levels of sociability and are frequently used as a comparative strain against BTBR mice [37,38]. In the present study, we investigated the effect of 5-AIQ on Treg cells and expression of proinflammatory cytokines in BTBR and C57 mice. We hypothesize that 5-AIQ treatment could be used to ameliorate the immune abnormalities of autism

## 2. Material Methods

### 2.1. Reagents and Antibodies

5. -AIQ was obtained from Matrix Scientific (Columbia, SC, USA. Roswell Park Memorial Institute). RPMI 1640 medium was purchased from Sigma-Aldrich (St. Louis, MO, USA). Antibodies to FOXP3 (#SC-130666), Helios (#SC-390357), GATA3 (#SC-268), and IL-17A (#SC-374218) were purchased from Santa Cruz Biotech, (Dallas, TX, USA). GolgiStop was purchased from BD Biosciences (San Diego, CA, USA). Conjugated phycoerythrin (PE), fluoro-isothiocyanate (FITC), PE/Dazzle 594, allophycocyanin (APC). APC anti-CD4 (#100412), FITC anti-CD4 (#100510), APC anti-CXCR6 (#151106), FITC anti-CXCR6 (#151107), PE anti-FOXP3 (#126404), PE anti-Helios (#137206), APC anti-GATA3 (#653806), PE anti-IL-17A (#506903), PE anti-IL-9 (#514104), and APC anti-IL-10 (#505010) monoclonal antibodies, red blood cell lysis buffer, fixation buffer, and intracellular staining permeabilization buffer were all obtained from BioLegend (San Diego, CA, USA). TRIzol reagent was purchased from Invitrogen (Carlsbad, CA, USA). SYBR Green and High-Capacity cDNA reverse transcription kit were purchased from Applied Biosystems (Foster City, CA, USA). Primers were synthesized by GenScript (Piscataway, NJ, USA). Nitrocellulose membranes were obtained from Bio-Rad Laboratories (Hercules, CA, USA). Western blot chemiluminescence kit was purchased from Millipore (Billerica, MA, USA).

### 2.2. Animals

Male BTBR and C57 mice aged 10–12 weeks were purchased from the Jackson Laboratory (Bar Harbor, ME, USA). Mice were housed in a room at the Animal Facility of King Saud University, in which the temperature (22 °C ± 1 °C) and relative humidity were controlled with a 12-h light/dark cycle (07:00 h lights on). The mice were allowed free access to water and food ad libitum. Animal experiments were started after an acclimation period of 7 days. All animal experiments were conducted according to the National Institutes of Health guidelines for the care and use of animals in research and were approved by the King Saud University Animal Ethics Committee (Approval number KSU-SE-18-27).

### 2.3. Drug Administration

To explore the effect of 5-AIQ treatment, the animals were divided as follows: C57 mice receiving saline alone by (intraperitoneal, i.p.) injection served as the control group; C57 mice treated with 5-AIQ (1.5 mg/kg, i.p); BTBR mice receiving saline alone by i.p. injection; and BTBR mice treated with 5-AIQ (1.5 mg/kg, i.p). Treatments were administered for 10 days. The volume of drug administered to each mouse was given based on individual body weight. The dose of 5-AIQ was selected based on previous studies [31,33,39,40].

### 2.4. Qualitative Intracellular Cytokine and Transcription Factor Detection by Flow Cytometry

We used flow cytometry analysis to assess the production of FOXP3, Helios, GATA3, IL-9, IL-17A, and IL-10 by CXCR6+ and CD4+ T cells. Briefly, splenocytes were incubated with phorbol 12-myristate 13-acetate (PMA)/ionomycin (Sigma-Aldrich) for 4 h in the presence of brefeldin-A (GolgiPlug, BD Biosciences), which prevents the transport of cytokines and transcription factors out of the cell [12,33,41]. Cells were washed and surface stained for CD4, and CXCR6 surface receptors (BioLegend, San Diego, CA, USA). After permeabilization and fixation (BioLegend), the cells were stained with intracellular cytokines (anti-IL-9, anti-IL-10, and anti-IL-17A; BioLegend) and transcription factors (anti-FOXP3, anti-GATA3, and anti-Helios; BioLegend). The proportions of CXCR6+FOXP3+, CXCR6+Helios+, CD4+GATA3+, CD4+IL-9+, CXCR6+IL-10+, and CD4+IL-17A+ cells were acquired via a FC 500 flow cytometer and analyzed using CXP software (Beckman Coulter, Indianapolis, IN, USA).

### 2.5. Reverse Transcriptase Quantitative PCR (RT-qPCR)

Total RNA was isolated from the brain using TRIzol reagent (Invitrogen, Carlsbad, CA, USA), and the RNA concentration quantified (NanoDrop, Thermo Scientific, Waltham, MA, USA). cDNA was synthesized, and then amplified using SYBR Green PCR mix (Applied Biosystems) as previously described [12,42]. The specific primer sequences for FOXP3, Helios, GATA3, IL-9, IL-10, IL-17A and GAPDH are listed as follows: FOXP3 Forward, 5′-CTGGACAACCCAGCCATGAT-3′ and Reverse, 5′-ACATTGATCCCAGGTGGCAG-3′; Helios Forward, 5′-CTTCCATAGCCAGAGCGAGG-3′ and Reverse, 5′-AGTGGGGATAGGGAAGGCAT-3′; GATA3 Forward, 5′-GGAGTCTCCAAGTGTGCGAA-3′ and Reverse, 5′-TGGAATGCAGACACCACCTC-3′; IL-9 Forward, 5′-ACTGAGTTCCAGACTCCCGT-3′ and Reverse, 5′-CAGTTGGGACGGAGAGACAC-3′; IL-10 Forward, 5′-TAAGGCTGGCCACACTTGAG-3′ and Reverse, 5′-GTTTTCAGGGATGAAGCGGC-3′; IL-17A Forward, 5′-TCATCCCTCAAAGCTCAGCG-3′ and Reverse, 5′-TTCATTGCGGTGGAGAGTCC-3′; GAPDH Forward, 5′-TGACCACAGTCCATGCCATC-3′ and Reverse, 5′-CTCAGATGCCTGCTTCACCA-3′. The relative expression of FOXP3, Helios, GATA3, IL-9, IL-10 and IL-17A was normalized to GAPDH and calculated according to the 2−ΔΔC(t) method.

### 2.6. Western Blotting

Total protein was extracted from mouse brain tissue, and quantitation was performed by direct detect spectroscopy (EMD Millipore). Briefly, protein samples (40 μg) were separated via 10% SDS-PAGE and transferred to Polyvinylidene fluoride (PVDF) membrane (Bio-Rad, Hercules, CA) as previously reported (Ansari et al., 2017). Membranes were incubated with primary antibodies against FOXP3, Helios, GATA3, and IL-17A overnight at 4 °C and subsequently incubated for 2 h with HRP-conjugated secondary antibody (Santa Cruz Biotech, Dallas, TX, USA) at room temperature. The FOXP3, Helios, GATA3, IL-17A and β-actin bands were visualized by enhanced chemiluminescence HRP substrate (Millipore Corporation, Burlington, MA, USA), and their intensity was quantified against the β-actin band used as a loading control [42].

### 2.7. Statistical Analysis

All data were expressed as mean ± standard deviation (SD). The data were analyzed using two-way ANOVA followed by Tukey’s post-hoc test corrected for multiple comparisons. Statistical analyses were carried out using GraphPad Prism (GraphPad Software, San Diego, CA, USA). A *p*-value of <0.05 was considered significant.

## 3. Results

### 3.1. Treatment with 5-AIQ Upregulates Treg Cells in BTBR Mice

**Figure 1:**
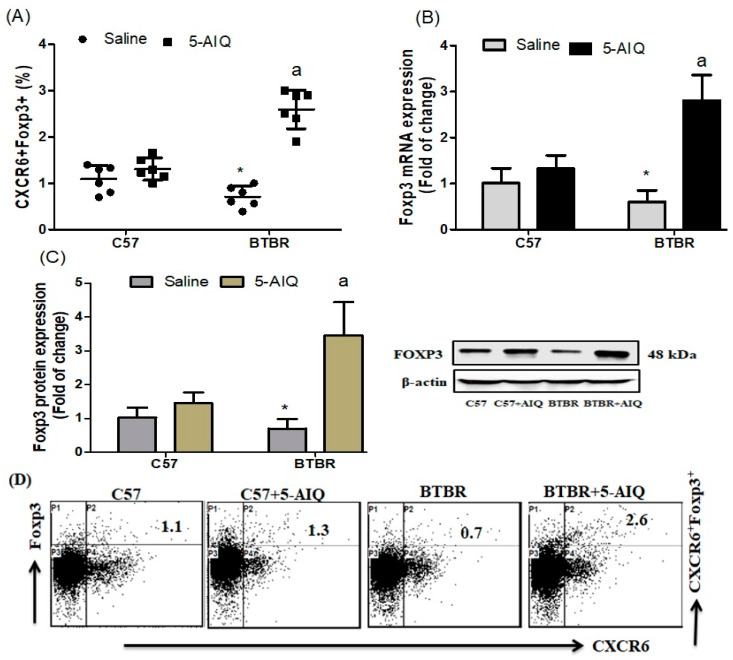
Effects of 5-AIQ on FOXP3 expression in C57 and BTBR mice. (**A**) Flow cytometry analysis of intracellular FOXP3-producing CXCR6+ T cells from mouse spleens (Strain effect, F(1,20) = 13.39, *p* < 0.0016; Treatment effect, F(1,20) = 71.37, *p* < 0.0001, Treatment x Strain effect, F(1,20) = 45.01, *p* < 0.0001); (**B**) Reverse Transcriptase Quantitative PCR (RT-qPCR) analysis of levels of FOXP3 mRNA from mouse brain tissue (Strain effect, F(1,20) = 12.52, *p* < 0.0021; Treatment effect, F(1,20) = 68.44, *p* < 0.0001, Treatment x Strain effect, F(1,20) = 38.81, *p* < 0.0001) and (**C**) Western blotting analysis of levels of FOXP3 protein from mouse brain tissue (Strain effect, F(1,20) = 13.48, *p* < 0.0015; Treatment effect, F(1,20) = 48.34, *p* < 0.0001, Treatment x Strain effect, F(1,20) = 26.23, *p* < 0.0001). (**D**) Cells were gated on forward-side scatter dot plots; the dot plots represent the percentages of CXCR6+FOXP3+ cells. Bar graphs represent the percentage of CXCR6+FOXP3+ population. The control C57 and BTBR mice received saline intraperitoneal injection. Treated BTBR and C57 mice received 5-AIQ (1.5 mg/kg) via intraperitoneal injection once daily for ten days. The results are shown as the means ± SD, *n* = 6, each group; * *p* < 0.05 compared with saline-treated C57 mice; a *p* < 0.05 compared with saline-treated BTBR mice.

To evaluate the therapeutic potential of 5-AIQ administration in the BTBR mouse model of autism, we first investigated the effect of 5-AIQ on the FOXP3-producing CXCR6+ cells in the spleen. 5-AIQ-treated BTBR mice had an increased percentage of FOXP3-producing CXCR6+ cells compared with those from saline-treated BTBR mice (Figure 1A). Using RT-qPCR and western blotting, we investigated the effect of 5-AIQ on expression levels of FOXP3 mRNA and protein in brain tissue. FOXP3 mRNA and protein expression levels were upregulated in 5-AIQ-treated BTBR mice compared with those in saline-treated BTBR mice (Figure 1B,C). In the present study, 5-AIQ treatment in BTBR mice significantly upregulated generation of Treg cells.

**Figure 2:**
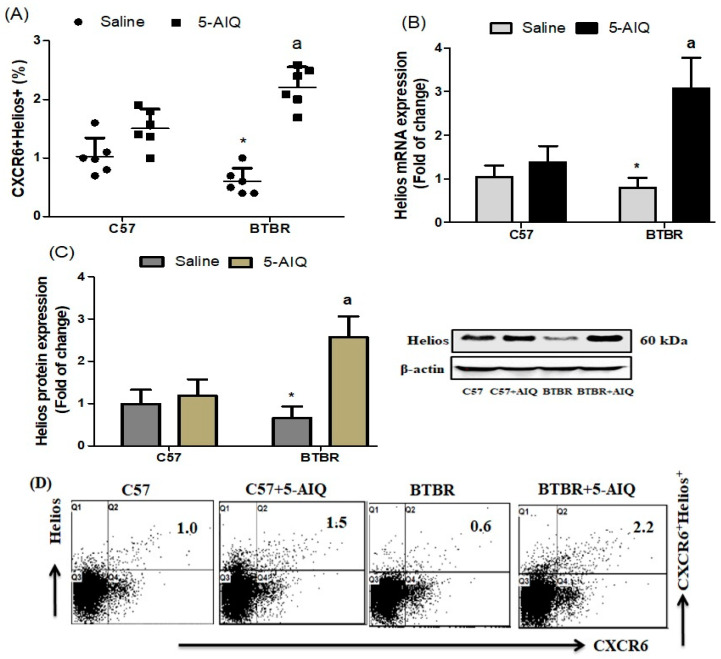
Effects of 5-AIQ on expression of Helios in C57 and BTBR mice. (**A**) Flow cytometry analysis of intracellular Helios-producing CXCR6+ T cells from mouse spleens tissue (Strain effect, F(1,20) = 1.209, *p* < 0.05; Treatment effect, F(1,20) = 69.48, *p* < 0.0001, Treatment x Strain effect, F(1,20) = 20.59, *p* < 0.0002); (**B**) RT-qPCR analysis of levels of Helios mRNA from mouse brain tissue (Strain effect, F(1,20) = 16.41, *p* < 0.0026; Treatment effect, F(1,20) = 53.77, *p* < 0.0001, Treatment x Strain effect, F(1,20) = 29.68, *p* < 0.0001) and (**C**) Western blotting analysis of levels of Helios protein from mouse brain tissue (Strain effect, F(1,20) = 11.79, *p* < 0.0026; Treatment effect, F(1,20) = 47.47, *p* < 0.0001, Treatment x Strain effect, F(1,20) = 31.20, *p* < 0.0001). (**D**) Cells were gated on forward-side scatter dot plots; the dot plots represent the percentages of CXCR6+Helios+ cells. Bar graphs represent the percentage of the CXCR6+Helios+ population. The control C57 and BTBR mice received saline intraperitoneal injection. Treated BTBR and C57 mice received 5-AIQ (1.5 mg/kg) via intraperitoneal injection once daily for ten days. The results are shown as the means ± SD, *n* = 6, each group; * *p* < 0.05 compared with saline-treated C57 mice; a *p* < 0.05 compared with saline-treated BTBR mice.

To further reveal the effect of 5-AIQ in BTBR mice, we evaluated Helios expression by CXCR6+ spleen cells. BTBR mice displayed a significant reduction in the number of Helios-producing CXCR6+ cells, which was significantly increased by 5-AIQ treatment (Figure 2A). The mRNA and protein levels of Helios in the brain tissue of BTBR mice were decreased in comparison with those of C57 mice and were significantly upregulated in brain tissue of 5-AIQ-treated BTBR mice (Figure 2C,D). These results reveal the effect of 5-AIQ on Helios expression in the autistic mouse model and open a new mechanism of action of 5-AIQ.

**Figure 3:**
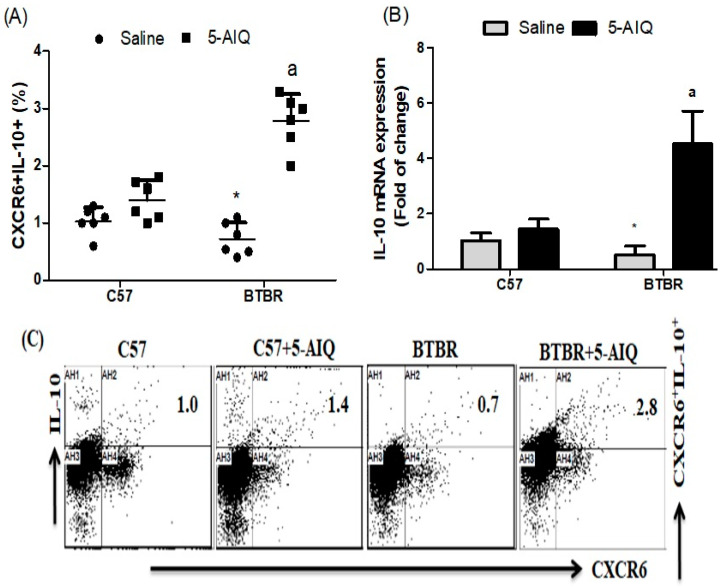
Effects of 5-AIQ on expression of IL-10 in C57 and BTBR mice. (**A**) Flow cytometry analysis of intracellular IL-10-producing CXCR6+ T cells from mouse spleens (Strain effect, F(1,20) = 14.27, *p* < 0.0012; Treatment effect, F(1,20) = 73.62, *p* < 0.0001, Treatment x Strain effect, F(1,20) = 35.61, *p* < 0.0001) and (**B**) RT-qPCR analysis of levels of IL-10 mRNA from mouse brain tissue (Strain effect, F(1,20) = 23.33, *p* < 0.0001; Treatment effect, F(1,20) = 68.56, *p* < 0.0001, Treatment x Strain effect, F(1,20) = 45.45, *p* < 0.0001). **(C)** Cells were gated on forward-side scatter dot plots; the dot plots represent the percentages of CXCR6+IL-10+ cells. Bar graphs represent the percentage of CXCR6+IL-10+ population. The control C57 and BTBR mice received saline intraperitoneal injection. Treated BTBR and C57 mice received 5-AIQ (1.5 mg/kg) via intraperitoneal injection once daily for ten days. The results are shown as the means ± SD, *n* = 6, each group; * *p* < 0.05 compared with saline-treated C57 mice; a *p* < 0.05 compared with saline-treated BTBR mice.

To investigate whether 5-AIQ played an anti-inflammatory effect in BTBR mice, we detected the production/expression of anti-inflammatory cytokines in spleen and brain tissues. The number of IL-10-producing CXCR6+ cells was increased in 5-AIQ-treated BTBR mice compared with levels from the spleen of saline-treated BTBR mice (Figure 3A). To further investigate whether 5-AIQ could upregulate the activities of Treg cells, we assessed the level of IL-10 mRNA in the brain of mice. Treatment with 5-AIQ significantly upregulated the level of IL-10 mRNA, which was related to Treg cell activity (Figure 3B). Therefore, treatment with 5-AIQ increased expression of IL-10 and could have a potent immunomodulatory potential for the treatment of autism.

### 3.2. Effects of 5-AIQ on GATA3 Transcription Factor

**Figure 4:**
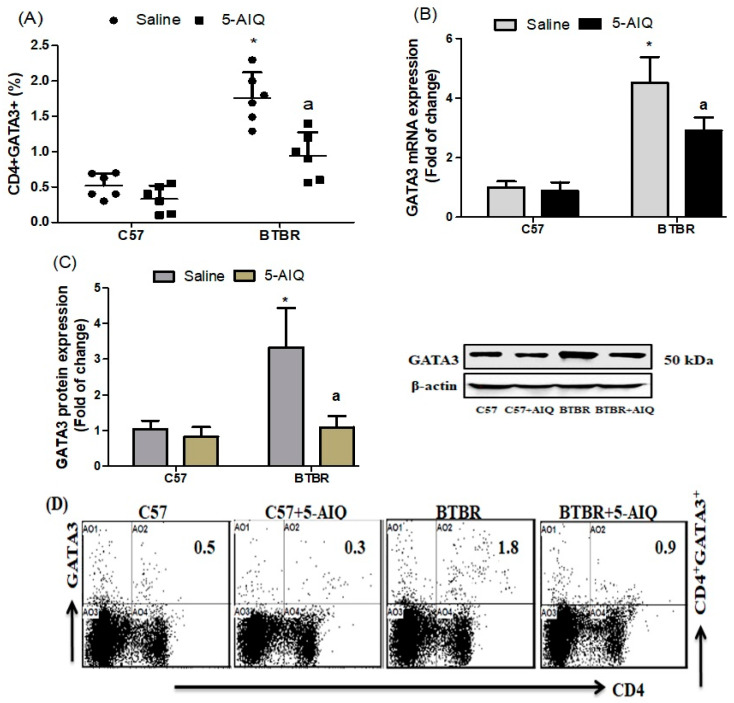
Effects of 5-AIQ on expression of GATA3 in C57 and BTBR mice. (**A**) Flow cytometry analysis of intracellular GATA3-producing CD4+ T cells from mouse spleens (Strain effect, F(1,20) = 67.84, *p* < 0.0001; Treatment effect, F(1,20) = 20.07, *p* < 0.0002, Treatment x Strain effect, F(1,20) = 7.728, *p* < 0.0116); (**B**) RT-qPCR analysis of levels of GATA3 mRNA from mouse brain tissue (Strain effect, F(1,20) = 171.5, *p* < 0.0001; Treatment effect, F(1,20) = 16.70, *p* < 0.0006, Treatment x Strain effect, F(1,20) = 12.49, *p* < 0.0021) and (**C**) Western blotting analysis of levels of GATA3 protein from mouse brain tissue (Strain effect, F(1,20) = 27.36, *p* < 0.0001; Treatment effect, F(1,20) = 24.79, *p* < 0.0001, Treatment x Strain effect, F(1,20) = 17.18, *p* < 0.0005). (**D**) Cells were gated on forward-side scatter dot plots; the dot plots represent the percentages of CD4+GATA3+ cells. Bar graphs represent the percentage of CD4+GATA3+ population. The control C57 and BTBR mice received saline intraperitoneal injection. Treated BTBR and C57 mice received 5-AIQ (1.5 mg/kg) via intraperitoneal injection once daily for ten days. The results are shown as the means ± SD, *n* = 6, each group; * *p* < 0.05 compared with saline-treated C57 mice; a *p* < 0.05 compared with saline-treated BTBR mice.

We examined the expression of GATA3 in CD4+ T cells from BTBR and C57 mice. 5-AIQ-treated BTBR mice markedly downregulated the expression of GATA3 in CD4+ T cells as compared with that from spleen cells in untreated BTBR mice (Figure 4A). There was also a significant decrease in GATA3 mRNA and protein expression levels in the brain tissue of 5-AIQ-treated BTBR mice (Figure 4B,C). Therefore, 5-AIQ administration downregulated GATA3 transcription factor signaling in BTBR mice.

### 3.3. 5-AIQ Treatment Downregulates Th9 Cells in BTBR Mice

**Figure 5:**
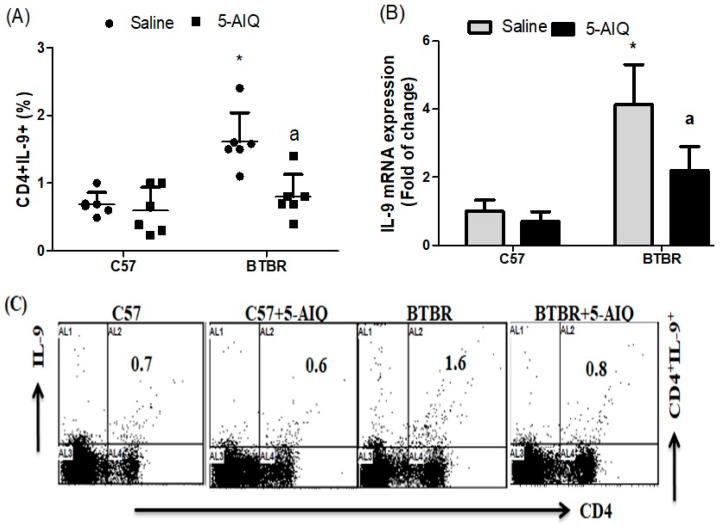
Effects of 5-AIQ on expression of IL-9 in C57 and BTBR mice. (**A**) Flow cytometry analysis of intracellular IL-9-producing CD4+ T cells from mouse spleens (Strain effect, F(1,20) = 17.33, *p* < 0.0005; Treatment effect, F(1,20) = 11.3, *p* < 0.0031, Treatment x Strain effect, F(1,20) = 7.31, *p* < 0.0147) and (**B**) RT-qPCR analysis of levels of IL-9 mRNA from mouse brain tissue (Strain effect, F(1,20) = 251.9, *p* < 0.0001; Treatment effect, F(1,20) = 26.00, *p* < 0.0001, Treatment x Strain effect, F(1,20) = 16.13, *p* < 0.0007). (**C**) Cells were gated on forward-side scatter dot plots; the dot plots represent the percentages of CD4+IL-9+ cells. Bar graphs represent the percentage of CD4+IL-9+ population. The control C57 and BTBR mice received saline intraperitoneal injection. Treated BTBR and C57 mice received 5-AIQ (1.5 mg/kg) via intraperitoneal injection once daily for ten days. The results are shown as the means ± SD, *n* = 6, each group; * *p* < 0.05 compared with saline-treated C57 mice; a *p* < 0.05 compared with saline-treated BTBR mice.

The numbers of IL-9-producing CD4+ T cells in the spleen of 5-AIQ-treated BTBR mice were significantly lower compared with those in saline-treated BTBR mice (Figure 5A). The relative levels of IL-9 mRNA were also significantly reduced in the brain of BTBR mice compared with those in saline-treated BTBR mice (Figure 5B). Taken together, these results indicate that 5-AIQ effectively decreases Th9 cell numbers in BTBR mice.

### 3.4. Effects of 5-AIQ Treatment on Th17 Cells

**Figure 6:**
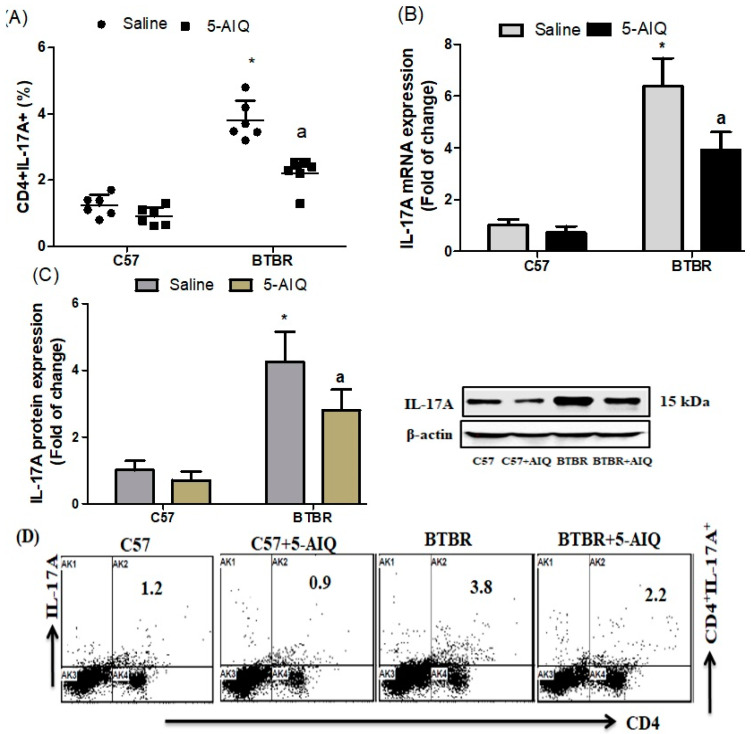
Effects of 5-AIQ on expression of IL-17A in C57 and BTBR mice (**A**) Flow cytometry analysis of intracellular IL-17A-producing CD4+ T cells from mouse spleens (Strain effect, F(1,20) = 120.8, *p* < 0.0001; Treatment effect, F(1,20) = 30.52, *p* < 0.0001, Treatment x Strain effect, F(1,20) = 13.54, *p* < 0.0115); (**B**) RT-qPCR analysis of levels of IL-17A mRNA from mouse brain tissue (Strain effect, F(1,20) = 62.22, *p* < 0.0001; Treatment effect, F(1,20) = 14.47, *p* < 0.0011, Treatment x Strain effect, F(1,20) = 7.797, *p* < 0.0112) and (**C**) Western blotting analysis of levels of IL-17A protein from mouse brain tissue (Strain effect, F(1,20) = 127.8, *p* < 0.0001; Treatment effect, F(1,20) = 13.8, *p* < 0.0014, Treatment x Strain effect, F(1,20) = 5.909, *p* < 0.0246). (**D**) Cells were gated on forward-side scatter dot plots; the dot plots represent the percentages of CD4+IL-17A+ cells. Bar graphs represent the percentage of CD4+IL-17A+ population. The control C57 and BTBR mice received saline intraperitoneal injection. Treated BTBR and C57 mice received 5-AIQ (1.5 mg/kg) via intraperitoneal injection once daily for ten days. The results are shown as the means ± SD, *n* = 6, each group; * *p* < 0.05 compared with saline-treated C57 mice; a *p* < 0.05 compared with saline-treated BTBR mice.

To gain a deeper understanding of the mechanism associated in the neuroprotective effect of 5-AIQ on BTBR mice, we studied the effect of 5-AIQ on Th17 cells, which play an important role in the neurodevelopment of ASD. We observed a decrease in the number of IL-17A-producing CD4+ spleen cells in 5-AIQ-treated BTBR mice compared with those from BTBR saline-treated mice (Figure 6A). The expression levels of IL-17A mRNA and protein was highest in the brain tissue of BTBR compared with those in C57 mice. However, IL-17A expression was significantly decreased with 5-AIQ treatment in BTBR mice (Figure 6B,C). Taken together, these results indicate that 5-AIQ could protect against the development of ASD in BTBR mice.

## 4. Discussion

Inhibition of PARP-1 has been shown to prevent neurobehavioral and neurochemical abnormalities [32]. PARP-1 inhibition decreased brain infarction and neutrophil infiltration after transient focal cerebral ischemia [43] and can protect against traumatic injury and decrease nitric oxide production [44]. A previous study also demonstrated that inhibition of PARP-1 reduced motor deficits as well as improved behavioral assessment [45]. Neuroprotective effects for PARP-1 inhibition have also been presented [46]. Therapeutics that ameliorate the core symptoms of ASD remain unavailable and are sorely needed due to the increasingly recognized high prevalence of the disorder. Recently, we demonstrated that treatment with 5-AIQ markedly attenuated repetitive behavior and enhanced social interaction, which could indicate that correct immune functioning had been restored [33]. In the present study, we sought to determine how the effect of 5-AIQ on Treg/Th17 cells could help elucidate potential therapeutic indications for the treatment of ASD.

Several studies have evaluated the role of Treg cells in immune-mediated inflammatory diseases as an important contributing feature [47,48]. Furthermore, Treg cells are diminished in neuro-immunological diseases and this reduction has been associated with upregulation of other cell types [49,50]. Treg cells play a critical function in immunological self-tolerance to prevent severe systemic inflammation and their deficiency contributes to ASD and autoimmune disease [51,52]. Treg deficiency has also been reported in children with autism [14]. In our recent studies, we found that decreased levels of Treg cells potentially contribute to the ASD-like behavior in BTBR mice [12,53]. Previous results have identified the role of Helios in Treg differentiation [54]. In accordance with previous reports, FOXP3^+^ Tregs highly expressed CCR4, CCR5, CCR6, CXCR3, and CXCR6 [55]. We have also reported that immune dysregulation in ASD individuals is associated with decreased levels of Treg cells [11,56]. FOXP3 is a key transcription factor for Treg cells. In the present report study, FOXP3- and Helios-producing CXCR6+ cells in 5-AIQ-treated BTBR mice were significantly higher than those in untreated BTBR mice. We also found that 5-AIQ treatment markedly increased the mRNA and protein expression of FOXP3 and Helios in the brain tissue of BTBR mice. Importantly, our findings suggest that enhancing Treg cells by treatment with 5-AIQ may provide beneficial effects in ASD, which could represent a promising approach for ASD pharmacotherapy development.

The anti-inflammatory cytokine IL-10 has been shown to improve the neuronal threshold of vulnerability to ischemic damage in the CNS [57]. IL-10 is also known to inhibit glutamate-mediated neuronal apoptosis [58], and IL-10 production is known to be significantly lower in BTBR mice than in C57 mice [37]. However, the mechanisms underlying the neuroprotective activity of IL-10 are not fully understood. We investigated how 5-AIQ produced an anti-inflammatory effect in BTBR mice and assessed the expression of important IL-10 anti-inflammatory cytokines in mouse spleen and brain tissue. The expression level of IL-10 was significantly decreased in untreated BTBR mice, whereas this was significant increase in 5-AIQ-treated BTBR mice. We propose that 5-AIQ has neuroprotective effects against ASD because of the ability of 5-AIQ to increase the expression of IL-10 in BTBR mice.

BTBR mice have been reported to have an increased number of Th2 cells, which induce B cells to produce immunoglobulin [59], and a higher level of serum IgG has been demonstrated in BTBR mice [60]. In addition, expression of GATA3 has been shown to be associated in the development of the CNS [61]. This higher expression of GATA3 of ASD individuals could be useful as a disease biomarker [11,62]. We determined that expression of GATA3 in CD4+ spleen cells and brain tissue was significantly higher in untreated BTBR (autistic) mice, providing further evidence to support previous reports that GATA3 may be essential in the pathophysiology of ASD, possibly by causing abnormalities in the immune system [11,12,63]. We observed a marked reduction of GATA3-producing CD4+ T cells in 5-AIQ-treated BTBR mice when compared with those in saline-treated BTBR mice. There was also a decrease in expression of GATA3 mRNA and protein levels in 5-AIQ-treated BTBR mice. These results further confirmed the therapeutic effect of 5-AIQ on BTBR mice.

IL-9 mediates several types of inflammation in autoimmune diseases [64,65]. A previous study found that IL-9 production was significantly increased in brain tissue [66]. In addition, IL-9 has been implicated as a mediator of Th17-driven inflammatory diseases [66]. IL-9 promotes Th17 cell migration into the CNS via the CC chemokine ligand-20 (CCL20) produced by astrocytes [23]. Here, treatment with 5-AIQ decreased the abundance of IL-9-producing CD4+ T cells, as well as expression of mRNA in spleen cells and brain tissue of BTBR mice. Taken together, these data suggest that treatment with 5-AIQ is able to restore the both production and expression of IL-9, suggesting that 5-AIQ could be a therapeutic candidate for restoring neuroimmune dysfunction in ASD.

Whole brains were utilized for different molecular analyses without perfusion in this study. T cells from the brain tissue were not isolated to evaluate their exact role in inflammatory/anti-inflammatory signaling. Therefore, differences in molecular parameters observed in treated and untreated groups could originate both from immune cells such as T cells and from neuronal cells such as microglial cells in the brain. Further, it should be kept in mind that findings in whole-brain gene expression and protein levels might be different in specific brain areas this can provide more relevant information with regards to ASD. These are the limitations of this study.

Th17 cells have been recognized as inducers of autoimmunity and their exaggerated functions causes pathogenesis of inflammatory and autoimmune disorders [67]. Th17 cells have also been suggested to have an important role in ASD. Elevated levels of IL-17A have been detected in children with autism [68,69]. A genome-wide copy number variant analysis identified IL17A as one of many genes enriched in autistic patients [70]. As shown in a recent study, the expression of Th17 cells were higher in ASD [11,71], and a direct association between Th17 and disease severity in children with ASD has also been reported [72]. We observed a significant reduction in the abundance of IL-17A-producing CD4+ T cells in the spleens of 5-AIQ-treated BTBR mice. Moreover, 5-AIQ treatment significantly downregulated expression of IL-17A mRNA and protein in the brain tissue of BTBR mice. These findings suggest that inhibition of IL-17A production via treatment with 5-AIQ could be helpful in treating the behavioral deficits in ASD. Therefore, the efficacy of 5-AIQ in reducing IL-17A expression is a novel finding and adds to the potential therapeutic indications for treating ASD with 5-AIQ

## 5. Conclusions

Our study has provided several pieces of evidence that address the pivotal role of PARP-1 inhibition in the mouse autism model. We have demonstrated that 5-AIQ had a therapeutic effect on BTBR mice, which was associated with modulation of Treg and Th17 cells. We also confirmed the effectiveness of 5-AIQ, emphasizing the importance of neuroimmune function as a target that deserves to be investigated in preclinical studies of anti-inflammatory therapeutic approaches in ASD. Furthermore, our study highlighted a promising experimental strategy to evaluate new molecular targets possibly involved in the development of neuroimmune dysfunctions in ASD.

## Data Availability

The authors confirm that all data underlying the findings are fully available without restriction. All relevant data are within the paper.

## Abbreviations

Autism spectrum disorder (ASD); Forkhead box P3 (Foxp3); C-X-C chemokine receptor type 6 (CXCR6); Cluster of differentiation 4 (CD4); T helper (Th); GATA binding protein 3 (GATA3); 5-Aminoisoquinolinone (5-AIQ); Interleukin 10 (IL-10), BTBR T+ Itpr3tf/J (BTBR); Regulatory T (Treg); Central nervous system (CNS); Poly(ADP-ribose) polymerase-1 (PARP)-1; C57BL/6 (C57); Phycoerythrin (PE); allophycocyanin (APC); Fluoro-isothiocyanate (FITC); Messenger RNA (mRNA); Reverse transcription polymerase chain reaction (RT-PCR); Intraperitoneally (i.p.); Dimethyl sulfoxide (DMSO); CC chemokine ligand-20 (CCL20).
